# Revisiting Imagery in Psychopathology: Why Mechanisms Are Important

**DOI:** 10.3389/fpsyt.2019.00457

**Published:** 2019-07-05

**Authors:** Helen O’Shea, Aidan Moran

**Affiliations:** School of Psychology, University College Dublin, Dublin, Ireland

**Keywords:** motor imagery, intrusive imagery, obsessive compulsive disorder, post-traumatic stress disorder, motor simulation theory

Humans have an exceptional capacity to mentally simulate sensory information of stimuli that are not physically present. This high-level cognitive ability—known as mental imagery—is a multisensory process that exploits internal representations of perception and/or action in working memory (1). Imagery mechanisms are fundamental to the computational functioning of the brain, facilitating predictive processing (2) and guiding future behavior (3). If imagined prospective possibilities are grounded in appropriate mental representations, successful behavior may ensue (3), but if not, psychopathological tendencies may arise (e.g., suicidal “flashforwards”) (4). Whether used explicitly (consciously and voluntarily) or implicitly (unconsciously and/or involuntarily), imagery can simulate (absent) sensorimotor information to improve memory and enhance skills (5). However, when imagery is intrusive or uncontrolled, it may signal psychopathology. Involuntary and/or distressing imagery plays a ubiquitous role in the etiology and maintenance of many mental disorders (6) such as OCD (7), PTSD (8), and suicidality (4). Dysfunctional imagery also contributes to maladaptive interpersonal patterns [e.g., Ref. (9)] and reduced capacity for a positive future-orientated outlook (10).

Although considerable progress has been made in understanding imagery processes in healthy populations, less is known about these in the clinical domain. Specifically, the mechanisms driving the development, maintenance, and/or treatment of dysfunctional imagery in psychopathology remain largely unresolved (11). So the present paper explains why research on imagery mechanisms is important in understanding certain clinical conditions. Drawing on research on motor imagery (MI), we highlight imagery-related mechanisms involved in clinical disorders with motor components (e.g., compulsions in OCD). It is beyond our scope to investigate imagery content in clinical disorders. Instead, we focus on plausible mechanisms underlying imagery processes in psychopathology. We begin by outlining key characteristics of MI before discussing possible mechanisms driving intrusive images in certain clinical conditions. Implications for treatment are also considered.

## Advances in the Science of Motor Imagery (MI)

MI is a dynamic mental process that involves the performance of actions in the mind while inhibiting motor output (12). It is characteristically accompanied by subjective visual and/or kinesthetic sensations—one “sees” and/or “feels” one’s body move (13). So patients’ MI experiences involve a realness that often results in more emotional arousal than would be experienced otherwise (14). Further, MI is associated with psychophysiological responses (15), which suggest active processing of motor representations. Heightened motorsensory, psychophysiological, and emotional information during MI may mask conceptual processing of events and increase the likelihood and frequency of intrusive imagery (16). Interestingly, psychophysiological responses can provide an adjunct measure of symptom severity in PTSD (17). The systematic use of MI is associated with changes at neural and behavioral levels (18). Such findings highlight a potential mechanism by which dysfunctional imagery might be maintained. If negative imagery continues over time, neural motor systems will be shaped accordingly and associated maladjusted behaviors strengthened. Finally, first-person kinesthetic MI (i.e., where we feel our own body perform the action) activates primary motor cortical networks and facilitates corticospinal excitability (19). The presence of such neural activity during MI strengthens a sense of body ownership and controlled agency (20). Accordingly, this imagery perspective may empower patients by providing them with a sense of control over their bodily experiences.

Motor simulation theory (MST; 12, 21) postulates that MI is functionally equivalent to the physical execution of action because the two movement types share brain systems and functional mechanisms [see Ref. (22)]. During MI, similar motor representations to those involved during action execution are activated, and a putative simulation mechanism enables offline/cognitive performance of the represented action (12). Thus, MI primes the motor system in anticipation of acting. The key difference between MI and executed action is that the former must employ inhibitory mechanisms to prevent overt movement (21). This theory is supported by evidence that MI and actual action rely on similar neuroanatomical structures and activate overlapping neural networks [see Ref. (23)]. However, the overlap is not perfect (24), and MI activates additional ventral neural circuits linking the prefrontal and parietal cortices—thought to facilitate conscious cognitive control (25). Cognitive control is important in imagery-related psychopathology particularly given the experienced realness of images (10). Without such control, patients may be susceptible to relapse. Imagery likely involves several levels of cognition from low-level unconscious simulation to high-level conscious MI and even meta-cognitive awareness. Interestingly, research demonstrates that compulsions or irresistible urges to act (as observed in OCD) are linked to impaired attentional capacity (7). Furthermore, interventions (e.g., cognitive behavioral therapy) that increase neural connectivity in the dorsolateral and ventrolateral prefrontal cortices (networks crucial for inhibitory control) are associated with greater resistance to compulsion (26). Accordingly, if used appropriately and with patients with good imagery ability, imagery-related treatments may strengthen activity in networks mediating conscious cognitive control.

Progress has also been made in understanding the specific cognitive systems supporting and constraining MI (22). Attentional systems are important during MI because images are formed and consciously manipulated in working memory. Supervisory attentional mechanisms bias the selection of specific goal-compatible action schemata held in long-term memory, integrating the various action components involved (12). Attentional systems also help to guide imagined actions towards completion, supervising lower-level (fast and automatic) cognitive simulation processes that anticipate sensory consequences of the action (12). Importantly, meta-cognitive processes (i.e., people’s knowledge and awareness of, and control over their own cognitive processes) (27) may supervise this entire process, ensuring control over the imagined action and disengagement of the simulation mechanism. So meta-cognitive/imagery processes may represent a valuable target for future imagery-related interventions because they may facilitate the cognitive control required to manage intrusive images [e.g., Ref. (28)]. Furthermore, meta-awareness of momentary attentional bias towards emotional information leads to greater regulation of subsequent behavior. By contrast, inadequate meta-awareness may prolong dysregulated attentional behavior (29). Another cognitive process underpinning MI is inhibition. Although MI and executed action share functional mechanisms, MI must engage inhibition to prevent overt movement (21). This inhibition can be implemented with minimal attentional effort at the action intention stage (19, 30) or by actively maintaining a balance between the production and inhibition of motor commands (31). The extent of attentional effort involved in inhibition has clinical relevance as it may alter the outcomes of the treatment; the frequency of, and/or control over, imaginal intrusions; or the patient’s experience of the treatment itself (32). Future research could fruitfully investigate the role of inhibitory processes in imagery-related disorder intervention. Recent studies show that “practice” at inhibiting intrusions through repeated experience of suppressing trauma may reduce the impact of unwanted memories through enhanced inhibitory control (33). Such practice seems particularly important in OCD, which is characterized by an overwhelming urge to perform compulsive acts (7). In summary, MI mechanisms involve attention, working memory, and long-term memory. The next section will relate such neurocognitive mechanisms of MI to intrusive imagery in clinical disorders.

## MI and Intrusive Negative Images

Intrusive imagery is an undesirable and involuntary experience of negative and/or distressing images invading conscious awareness (34). These images are typically persistent, detailed, and dynamic and involve not only visual imagery but also motor and autonomic arousal elements, many of which are unconscious (35). Intrusive imagery occurs across a range of clinical disorders, including depression and suicidality (11), OCD (36), and PTSD (8). Psychotherapeutic treatment for these difficulties often involves some form of imaginal exposure and/or imagery rescripting, often as part of cognitive behavioral regimes (37). Imagery rescripting involves altering toxic memories/representations to create, *via* imagery, an alternative (more beneficial) mental representation of an event (38). Although the vividness and/or frequency of intrusive imagery may decline after such treatment (39, 40), the mechanisms producing such change remain unclear (33, 37). This section will attempt to address this issue. Specifically, what is the role of MI in generating and driving (motor-related) intrusive imagery in clinical disorders and the positive treatment outcomes observed following imagery rescripting interventions (41)?

According to MST, MI primes the motor system in anticipation of action execution (21). Although MI is underpinned by many cognitive systems, it is the fast and unconscious simulation that seems to be its key mechanism (21). So intrusive negative images might take hold when conscious control is weakened. As continued priming of neural motor systems may induce brain plasticity and reorganization (18), MI may contribute to the maintenance of imagery-related psychopathology. Therefore, early intervention for clinical disorders with imagery dysfunction may be important (34). If imagery-related neural plasticity occurs to a similar extent in imagined as in physical execution of action, then where actions are inappropriate (e.g., in OCD), MI might offer a viable route to altering maladaptive behavioral patterns. For example, habitual compulsive acts “ensure the persistence of obsessions” in OCD (p. 49, 7). So MI training might assist in the control, alteration, or diversion of compulsive behavior *via* inhibitory processes and attentional control (28). The attentional mechanisms supporting motor simulation/imagery are also relevant to imagery rescripting interventions because during rescripting they may help to create and bias a competitive retrieval preference for a new, less harmful (motor) memory of a traumatic event (38). Regarding higher-level control, research indicates that most individuals have a capacity for metacognitive processing (29). Interventions focusing on meta-cognition in mental health treatments is not new (28), and some, for example, in the interpersonal therapy domain [e.g., Ref. (42)], have demonstrated improvements in life quality. However, consideration of meta-cognitive processes in *imagery-related* intervention has largely been neglected to date [e.g., Ref. (32)]. So far, imagery interventions like rescripting (43) have largely focused on altering the meaning of events and associated images/memories (40). However, as previously suggested, they might also have a potentially powerful influence on altering the (uncontrolled) overt and covert behaviors of patients. Although re-scripting may help to relieve the initial distress associated with negative images, its long-term use requires a deeper understanding of relevant imagery mechanisms. Overall, MI is a complex psychological construct, supported and constrained by several cognitive processes, and thus, its association with imagery dysfunction and clinical disorder requires nuanced exploration and application at both content and function levels.

## Concluding Comments

This article highlights recent theoretical progress in imagery research and identifies some possible MI mechanisms driving intrusive negative imagery experiences in psychopathology. MST provides a potentially valuable theoretical basis for investigating, understanding, and treating maladapted imagery (with motor components) across clinical disorders. The simulation mechanism driving MI—and the higher-order cognitive systems that support it—represent potential key targets for future intervention. Very often, imaginal exposure therapy and re-experiencing are associated with distress and high dropout rates, and preferred imagery techniques tend to involve imagery rescripting (39). Therefore, developing enhanced rescripting interventions that take into consideration some of the underlying imagery mechanisms outlined above (attentional, inhibitory, and meta-cognitive processes) offer promising future directions. In summary, the present opinion paper aims to encourage dialogue and promote knowledge transfer between cognitive psychological/neuroscientific and clinical domains.

## Author Contributions

Both authors contributed equally to this article.

## Conflict of Interest Statement

The authors declare that the research was conducted in the absence of any commercial or financial relationships that could be construed as a potential conflict of interest.
